# Supplementary material for: Association of Elite Sports Status with Gene Variants of Peroxisome Proliferator Activated Receptors and Their Transcriptional Coactivator
Source: Int J Mol Sci. 2019 Dec 25;21(1):162. doi: 10.3390/ijms21010162 (PMC6981913; doi:10.3390/ijms21010162)
Supplement: Supplementary file 1 [file ijms-21-00162-s001.zip › ijms-634848-proof-suppl/S2_Table.docx]

**S2 Table. Strengthening the Reporting of Observational Studies in Epidemiology (STROBE) checklist**

| **Authors** | **1** | **2** | **3** | **4** | **5** | **6** | **7** | **8** | **9** | **10** | **11** | **12** | **13** | **14** | **15** | **16** | **17** | **18** | **19** | **20** |
| --- | --- | --- | --- | --- | --- | --- | --- | --- | --- | --- | --- | --- | --- | --- | --- | --- | --- | --- | --- | --- |
| Ahmetov et al. 2006 [[1](#_ENREF_1)] | **Y** | **Y** | **Y** | **Y** | **N** | **Y** | **Y** | **N** | **N** | **N** | **Y** | **Y** | **N** | **Y** | **N** | **Y** | **Y** | **N** | **Y** | **Y** |
| Ahmetov et al. 2007 [[2](#_ENREF_2)] | **Y** | **Y** | **Y** | **Y** | **N** | **Y** | **Y** | **N** | **N** | **N** | **Y** | **Y** | **N** | **Y** | **Y** | **Y** | **Y** | **N** | **Y** | **Y** |
| Ahmetov et al. 2008 [[3](#_ENREF_3)] | **Y** | **Y** | **Y** | **Y** | **N** | **Y** | **Y** | **Y** | **Y** | **N** | **Y** | **Y** | **N** | **Y** | **Y** | **Y** | **Y** | **Y** | **Y** | **Y** |
| Ahmetov et al. 2009 [[4](#_ENREF_4)] | **Y** | **Y** | **Y** | **Y** | **N** | **Y** | **Y** | **N** | **N** | **N** | **Y** | **Y** | **N** | **Y** | **N** | **Y** | **Y** | **N** | **Y** | **Y** |
| Cieszczyk et al. 2011 [[5](#_ENREF_5)] | **Y** | **Y** | **Y** | **Y** | **N** | **Y** | **Y** | **N** | **N** | **N** | **Y** | **N** | **N** | **Y** | **Y** | **Y** | **Y** | **N** | **Y** | **Y** |
| Cocci et al. 2019 [[6](#_ENREF_6)] | **Y** | **Y** | **Y** | **Y** | **N** | **Y** | **Y** | **N** | **N** | **N** | **Y** | **Y** | **N** | **Y** | **Y** | **Y** | **Y** | **N** | **Y** | **Y** |
| Drozdovska et al. 2013 [[7](#_ENREF_7)] | **Y** | **Y** | **Y** | **Y** | **N** | **Y** | **Y** | **N** | **N** | **N** | **Y** | **Y** | **N** | **Y** | **N** | **N** | **Y** | **N** | **Y** | **Y** |
| Egorova et al. 2014 [[8](#_ENREF_8)] | **Y** | **Y** | **Y** | **Y** | **N** | **Y** | **Y** | **N** | **N** | **N** | **Y** | **Y** | **N** | **Y** | **Y** | **N** | **Y** | **N** | **Y** | **Y** |
| Eynon et al. 2010 [[9](#_ENREF_9)] | **Y** | **Y** | **Y** | **Y** | **N** | **Y** | **Y** | **Y** | **N** | **N** | **Y** | **Y** | **N** | **Y** | **Y** | **Y** | **Y** | **Y** | **Y** | **Y** |
| Eynon et al. 2011 [[10](#_ENREF_10)] | **Y** | **Y** | **Y** | **Y** | **N** | **Y** | **Y** | **N** | **N** | **N** | **Y** | **Y** | **Y** | **Y** | **Y** | **Y** | **Y** | **N** | **Y** | **Y** |
| Eynon et al. 2011 [[11](#_ENREF_11)] | **Y** | **Y** | **Y** | **Y** | **N** | **Y** | **Y** | **N** | **N** | **N** | **Y** | **N** | **N** | **Y** | **N** | **N** | **Y** | **Y** | **Y** | **Y** |
| Gineviciene et al. 2011 [[12](#_ENREF_12)] | **Y** | **Y** | **Y** | **N** | **N** | **Y** | **Y** | **N** | **N** | **N** | **Y** | **N** | **N** | **Y** | **N** | **N** | **Y** | **N** | **Y** | **Y** |
| Gineviciene et al. 2014 [[13](#_ENREF_13)] | **Y** | **Y** | **Y** | **Y** | **N** | **Y** | **Y** | **N** | **N** | **N** | **Y** | **Y** | **N** | **Y** | **N** | **Y** | **Y** | **N** | **Y** | **Y** |
| Gineviciene et al. 2016 [[14](#_ENREF_14)] | **Y** | **Y** | **Y** | **Y** | **N** | **Y** | **Y** | **N** | **N** | **N** | **Y** | **Y** | **N** | **Y** | **Y** | **Y** | **Y** | **N** | **Y** | **Y** |
| Gonzales Freire et al. 2009 [[15](#_ENREF_15)] | **Y** | **Y** | **Y** | **Y** | **N** | **Y** | **Y** | **N** | **N** | **N** | **Y** | **Y** | **N** | **Y** | **Y** | **Y** | **Y** | **N** | **Y** | **Y** |
| Grealy et al. 2015 [[16](#_ENREF_16)] | **Y** | **Y** | **Y** | **Y** | **N** | **Y** | **Y** | **N** | **N** | **N** | **Y** | **Y** | **N** | **Y** | **Y** | **Y** | **Y** | **N** | **Y** | **Y** |
| Lucia et al. 2005 [[17](#_ENREF_17)] | **Y** | **Y** | **Y** | **Y** | **N** | **Y** | **Y** | **N** | **N** | **N** | **Y** | **Y** | **N** | **Y** | **Y** | **Y** | **Y** | **N** | **Y** | **Y** |
| Maciejewska et al. 2011 [[18](#_ENREF_18)] | **Y** | **Y** | **Y** | **Y** | **N** | **Y** | **Y** | **Y** | **Y** | **N** | **Y** | **Y** | **N** | **Y** | **Y** | **Y** | **Y** | **Y** | **Y** | **Y** |
| Maciejewska et al. 2012 [[19](#_ENREF_19)] | **Y** | **Y** | **Y** | **Y** | **N** | **Y** | **Y** | **N** | **N** | **N** | **Y** | **N** | **N** | **N** | **N** | **N** | **Y** | **N** | **Y** | **Y** |
| Maciejewska et al. 2013 [[20](#_ENREF_20)] | **Y** | **N** | **Y** | **Y** | **N** | **Y** | **Y** | **N** | **N** | **N** | **Y** | **Y** | **N** | **Y** | **N** | **N** | **Y** | **N** | **Y** | **Y** |
| Maciejewska et al. 2014 [[21](#_ENREF_21)] | **Y** | **Y** | **Y** | **Y** | **N** | **N** | **Y** | **N** | **N** | **N** | **Y** | **Y** | **N** | **Y** | **Y** | **Y** | **Y** | **N** | **Y** | **Y** |
| Muniesa et al. 2010 [[22](#_ENREF_22)] | **Y** | **Y** | **Y** | **Y** | **N** | **Y** | **Y** | **N** | **N** | **N** | **Y** | **Y** | **N** | **Y** | **N** | **Y** | **Y** | **N** | **Y** | **Y** |
| Peplonska et al. 2017 [[23](#_ENREF_23)] | **Y** | **Y** | **Y** | **Y** | **N** | **Y** | **Y** | **N** | **N** | **N** | **Y** | **Y** | **N** | **Y** | **N** | **Y** | **Y** | **N** | **Y** | **Y** |
| Santiago et al. 2010 [[24](#_ENREF_24)] | **Y** | **Y** | **Y** | **Y** | **N** | **Y** | **Y** | **N** | **N** | **N** | **Y** | **Y** | **N** | **Y** | **N** | **Y** | **Y** | **Y** | **Y** | **Y** |
| Tsianos et al. 2010 [[25](#_ENREF_25)] | **Y** | **Y** | **Y** | **Y** | **N** | **Y** | **Y** | **N** | **N** | **N** | **Y** | **Y** | **N** | **Y** | **N** | **Y** | **Y** | **N** | **Y** | **Y** |
| Tural et al. 2014 [[26](#_ENREF_26)] | **Y** | **Y** | **Y** | **Y** | **N** | **Y** | **Y** | **N** | **N** | **N** | **Y** | **Y** | **N** | **Y** | **N** | **Y** | **Y** | **N** | **Y** | **Y** |
| Yvert et al. 2016 [[27](#_ENREF_27)] | **Y** | **Y** | **Y** | **Y** | **N** | **Y** | **Y** | **N** | **N** | **N** | **Y** | **Y** | **N** | **Y** | **N** | **Y** | **Y** | **N** | **Y** | **Y** |

**The numbers shown in column represents the items from modified STROBE checklist. The detailed description of each item is in supplementary table 1. Y = Yes, the item is present in article. N = No, the item is not present in the article.**

1. Ahmetov, I.I.; Mozhayskaya, I.A.; Flavell, D.M.; Astratenkova, I.V.; Komkova, A.I.; Lyubaeva, E.V., et al.: PPARα gene variation and physical performance in Russian athletes. *Eur J Appl Physiol97*:103-8(**2006)**. doi.

2. Ahmetov, I.I.; Astratenkova, I.V.; Rogozkin, V.A.: Association of a PPARD polymorphism with human physical performance. *Molecular Biology41*:776-80(**2007)**. doi.

3. Ahmetov, I.I.; Mozhayskaya, I.A.; Lyubaeva, E.V.; Vinogradova, O.L.; Rogozkin, V.A.: PPARG Gene polymorphism and locomotor activity in humans. *Bull Exp Biol Med146*:630-2(**2008)**. doi.

4. Ahmetov, I.I.; Williams, A.G.; Popov, D.V.; Lyubaeva, E.V.; Hakimullina, A.M.; Fedotovskaya, O.N., et al.: The combined impact of metabolic gene polymorphisms on elite endurance athlete status and related phenotypes. *Human Genetics126*:751-61(**2009)**. doi.

5. Cieszczyk, P.; Sawczuk, M.; Maciejewska, A.; Ficek, K.; Eider, A.: Variation in peroxisome proliferator activated receptor α gene in elite combat athletes. *European Journal of Sport Science11*:119-23(**2011)**. doi.

6. Cocci, P.; Pistolesi, L.; Guercioni, M.; Belli, L.; Carli, D.; Palermo, F.A.: Genetic Variants and Mixed Sport Disciplines: A Comparison among Soccer, Combat and Motorcycle Athletes. *Annals of Applied Sport Science7*:1-9(**2019)**. doi.

7. Drozdovska, S.B.; Dosenko, V.E.; Ahmetov, I.I.; Ilyin, V.N.: The association of gene polymorphisms with athlete status in Ukrainians. *Biology of Sport30*:163-7(**2013)**. doi.

8. Egorova, E.S.; Borisova, A.V.; Mustafina, L.J.; Arkhipova, A.A.; Gabbasov, R.T.; Druzhevskaya, A.M., et al.: The polygenic profile of Russian football players. *Journal of Sports Sciences32*:1286-93(**2014)**. doi.

9. Eynon, N.; Meckel, Y.; Sagiv, M.; Yamin, C.; Amir, R.; Sagiv, M., et al.: Do PPARGC1A and PPARα polymorphisms influence sprint or endurance phenotypes? *Scandinavian Journal of Medicine and Science in Sports20*(**2010)**. doi.

10. Eynon, N.; Alves, A.J.; Yamin, C.; Meckel, Y.: PPARA intron 1 A/C polymorphism and elite athlete status. *European Journal of Sport Science11*:177-81(**2011)**. doi.

11. Eynon, N.; Ruiz, J.R.; Meckel, Y.; Morán, M.; Lucia, A.: Mitochondrial biogenesis related endurance genotype score and sports performance in athletes. *Mitochondrion11*:64-9(**2011)**. doi.

12. Ginevičiene, V.; Pranckevičiene, E.; Milašius, K.; Kučinskas, V.: Gene variants related to the power performance of the Lithuanian athletes. *Central European Journal of Biology6*:48-57(**2011)**. doi.

13. Gineviciene, V.; Jakaitiene, A.; Tubelis, L.; Kucinskas, V.: Variation in the ACE, PPARGC1A and PPARA genes in Lithuanian football players. *European Journal of Sport Science14*:S289-S95(**2014)**. doi.

14. Gineviciene, V.; Jakaitiene, A.; Aksenov, M.O.; Aksenova, A.V.; Druzhevskaya, A.M.; Astratenkova, I.V., et al.: Association analysis of ACE, ACTN3 and PPARGC1A gene polymorphisms in two cohorts of European strength and power athletes. *Biology of Sport33*:199-206(**2016)**. doi.

15. Gonzalez-Freire, M.; Santiago, C.; Verde, Z.; Lao, J.I.; Olivan, J.; Gallego, F.G., et al.: Unique among unique. Is it genetically determined? *British Journal of Sports Medicine43*:307-9(**2009)**. doi.

16. Grealy, R.; Herruer, J.; Smith, C.L.E.; Hiller, D.; Haseler, L.J.; Griffiths, L.R.: Evaluation of a 7-gene genetic profile for athletic endurance phenotype in ironman championship triathletes. *PLoS ONE10*(**2015)**. doi.

17. Lucia, A.; Gomez-Gallego, F.; Barroso, I.; Rabadan, M.; Bandres, F.; San Juan, A.F., et al.: PPARGC1A genotype (Gly482Ser) predicts exceptional endurance capacity in European men. *J Appl Physiol (1985)99*:344-8(**2005)**. doi.

18. Maciejewska, A.; Sawczuk, M.; Cieszczyk, P.: Variation in the PPARalpha gene in Polish rowers. *J Sci Med Sport14*:58-64(**2011)**. doi.

19. Maciejewska, A.; Sawczuk, M.; Cieszczyk, P.; Mozhayskaya, I.A.; Ahmetov, II. The PPARGC1A gene Gly482Ser in Polish and Russian athletes. *J Sports Sci30*:101-13(**2012)**. doi.

20. Maciejewska-Karlowska, A.; Sawczuk, M.; Cieszczyk, P.; Zarebska, A.; Sawczyn, S.: Association between the Pro12Ala Polymorphism of the Peroxisome Proliferator-Activated Receptor Gamma Gene and Strength Athlete Status. *PLoS ONE8*(**2013)**. doi.

21. Maciejewska-Karlowska, A.; Hanson, E.D.; Sawczuk, M.; Cieszczyk, P.; Eynon, N.: Genomic haplotype within the Peroxisome Proliferator-Activated Receptor Delta (PPARD) gene is associated with elite athletic status. *Scand J Med Sci Sports24*:e148-55(**2014)**. doi.

22. Muniesa, C.A.; González-Freire, M.; Santiago, C.; Lao, J.I.; Buxens, A.; Rubio, J.C., et al.: World-class performance in lightweight rowing: Is it genetically influenced? A comparison with cyclists, runners and non-athletes. *British Journal of Sports Medicine44*:898-901(**2010)**. doi.

23. Peplonska, B.; Adamczyk, J.G.; Siewierski, M.; Safranow, K.; Maruszak, A.; Sozanski, H., et al.: Genetic variants associated with physical and mental characteristics of the elite athletes in the Polish population. *Scandinavian Journal of Medicine and Science in Sports27*:788-800(**2017)**. doi.

24. Santiago, C.; Ruiz, J.R.; Muniesa, C.A.; González-Freire, M.; Gómez-Gallego, F.; Lucia, A.: Does the polygenic profile determine the potential for becoming a world-class athlete? Insights from the sport of rowing. *Scandinavian Journal of Medicine and Science in Sports20*(**2010)**. doi.

25. Tsianos, G.I.; Evangelou, E.; Boot, A.; Carola Zillikens, M.; Van Meurs, J.B.J.; Uitterlinden, A.G., et al.: Associations of polymorphisms of eight muscle- Or metabolism-related genes with performance in Mount Olympus marathon runners. *Journal of Applied Physiology108*:567-74(**2010)**. doi.

26. Tural, E.; Kara, N.; Agaoglu, S.A.; Elbistan, M.; Tasmektepligil, M.Y.; Imamoglu, O.: PPAR-α and PPARGC1A gene variants have strong effects on aerobic performance of Turkish elite endurance athletes. *Molecular Biology Reports41*:5799-804(**2014)**. doi.

27. Yvert, T.; Miyamoto‐Mikami, E.; Murakami, H.; Miyachi, M.; Kawahara, T.; Fuku, N.: Lack of replication of associations between multiple genetic polymorphisms and endurance athlete status in Japanese population. *Physiological Reports4*(**2016)**. doi.
